# COVID-19 2022 update: transition of the pandemic to the endemic phase

**DOI:** 10.1186/s40246-022-00392-1

**Published:** 2022-06-01

**Authors:** Michela Biancolella, Vito Luigi Colona, Ruty Mehrian-Shai, Jessica Lee Watt, Lucio Luzzatto, Giuseppe Novelli, Juergen K. V. Reichardt

**Affiliations:** 1 Department of Biology, Tor Vergata University of Rome, Rome, Italy; 2 Department of Biomedicine and Prevention, Tor Vergata University of Rome, 00133 Rome, Italy; 3 Sheba Medical Center, Pediatric Hemato-Oncology, Edmond and Lilly Safra Children’s Hospital, Tel Hashomer 2 Sheba Road, 52621 Ramat Gan, Israel; 4 College of Public Health, Medical and Veterinary Sciences, James Cook University, Smithfield, QLD 4878 Australia; 5 Department of Haematology and Blood Transfusion, Muhimbili University of Health and Allied Sciences, Dar es Salaam, Tanzania; 6 University of Florence, Florence, Italy; 7 IRCCS Neuromed, Pozzilli, Isernia Italy; 8 Department of Pharmacology, School of Medicine, University of Nevada, Reno, NV USA; 9 Australian Institute of Tropical Health and Medicine, James Cook University, Smithfield, QLD 4878 Australia; 10 Department of Biomedicine and Prevention, School of Medicine and Surgery, Via Montpellier 1, 00133 Rome, Italy

## Abstract

COVID-19, which is caused by the SARS-CoV-2, has ravaged the world for the past 2 years. Here, we review the current state of research into the disease with focus on its history, human genetics and genomics and the transition from the pandemic to the endemic phase. We are particularly concerned by the lack of solid information from the initial phases of the pandemic that highlighted the necessity for better preparation to face similar future threats. On the other hand, we are gratified by the progress into human genetic susceptibility investigations and we believe now is the time to explore the transition from the pandemic to the endemic phase. The latter will require worldwide vigilance and cooperation, especially in emerging countries. In the transition to the endemic phase, vaccination rates have lagged and developed countries should assist, as warranted, in bolstering vaccination rates worldwide. We also discuss the current status of vaccines and the outlook for COVID-19.

## Introduction

By the end of December 2019, information began to circulate on an alarming form of pneumonia, of unknown etiology, that was afflicting the district of Wuhan, China. Two years later, it is of common knowledge that was the beginning of a pandemic, as declared by the World Health Organization (WHO) [1], triggered by the new Severe Acute Respiratory Syndrome Coronavirus-2 (SARS-CoV-2), the causative agent of Coronavirus Disease 2019 (COVID-19).

While facing the critical times of the manifestation of a "fourth wave,” amenable to the appearance of new variants [2–4], there has been exponential growth of new data. These data explore the genetics of the virus, the interaction with the host, as well as short-term and long-term clinical manifestations. Due to this, we believe there is need to provide an updated overview to uphold the commitment made in our latest Editorial [5].

In recent months, we globally experienced a rise in daily cases, contributing to a total of 527,971,809 cases and 6,284,871 deaths since the beginning of the pandemic (Johns Hopkins University, CSSE, accessed on 2022) [6]. Circulating variants have been supplanted by the new variant of concern (VOC) SARS-CoV-2 B.1.1.529 (Omicron) and its sub-variants [7, tracking website accessed on April 19, 2022], as SARS-CoV-2 BA.1 and BA.2, which are now dominant in the USA (Centers for Disease Control and Prevention. COVID Data Tracker. Atlanta, GA: US Department of Health and Human Services, CDC; https://covid.cdc.gov/covid-data-tracker, accessed on April 19, 2022) [8] and globally. Characterized by a greater ability to evade immune responses acquired through infection with a different strain [9] or through vaccination [10], this variant seems to be able to change the profile of current outbreak and, unlike in the previous waves, a higher rate of reinfections is reported [11, 12]. These early data, still under investigation, urge the scientific community toward the search for therapeutic and preventive tools that can counter this evolutionary mechanism.

In our review “COVID-19 one year into the pandemic: from genetics and genomics to therapy, vaccination, and policy” [13], we strongly claimed the determining role of vaccines as the most valuable aid to halt the spreading of SARS-CoV-2. We can now affirm that the increasing vaccination rate, with a total of 11.184.961.194 doses administered (Johns Hopkins University, CSSE, accessed on April 19, 2022) [6], is contributing to the containment of hospitalizations and deaths in the population affected by COVID-19 [14–16].

The importance of a homogeneous and universal distribution of vaccines is becoming more evident and incisive in hindering the appearance of new variants. In this regard, the disparities between advanced and developing countries seem to worsen, and this results in the inability to cope with new manifestations, as highlighted by the appearance of the SARS-CoV-2 B.1.1.529 variant [17].

## Origin and current state

COVID-19 was first reported in 2019 [18, 19]. It has now raged worldwide for more than 2 years, affecting every corner of our globe with no clear indication how the pandemic started. An intelligible understanding of the origins of this pandemic is critical to be better prepared in the future. We lament that a panel proposed by the WHO in 2021 to achieve this goal has not yet reached significant conclusions [20].

**Fig. 1:**
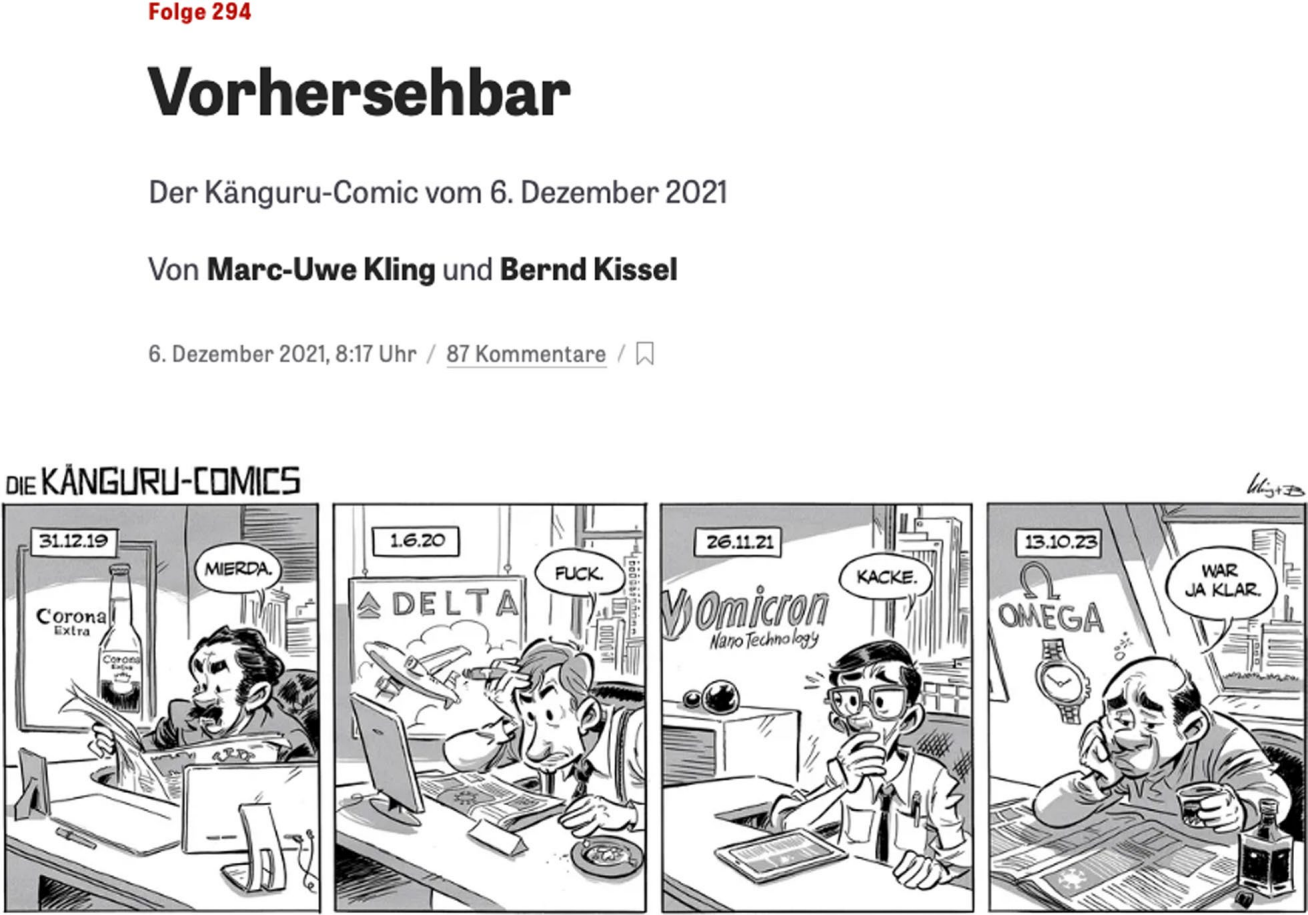
A comical view of the history of COVID-19. A few translations: “vorhersehbar” = predictable and “war ja klar” = obviously or sure or of course. Reproduced with permission (Mira Nagel)

While the pandemic is still raging, sprouts of hope have emerged that we may be transitioning into the endemic phase [21]. The pervasive Omicron variant, currently predominant, may lead to this course [22]. However, overly exuberant enthusiasm must be tempered by a sense of reality and concern for emerging countries [21, 23, 24]. Developed countries must remain vigilant and assist emerging countries in the fight against SARS-CoV-2, with the aim of detecting new variants of concern (Fig. 1), investigating animal reservoirs and sharing diagnostic tools, surveillance and therapies [25].

## Clinical manifestation of COVID-19 and post-acute (long) COVID-19

It is now known that COVID-19 is a multisystem condition that largely involves the respiratory system. It starts as an upper respiratory tract infection that subsequently affects the lungs and establishes, in the most severe cases, interstitial pneumonia (showing the diagnostic ground glass appearance, through CT investigation), severe respiratory failure, systemic inflammatory response and multi-organ dysfunction. Classic symptoms of the disease are listed as fever, asthenia, dry cough, nasal congestion and breathing difficulties.

Signs and symptoms, however, can affect several organs. Other systems can be involved, such as the central nervous system (hypo-anosmia, loss of the sense of taste, speech disturbances, dizziness, alterations of the consciousness and behavior, impaired walking and maintenance of upright position, impaired hearing and vision), the cardiovascular system (alterations in hemostasis, arrhythmia, heart failure), the gastrointestinal system (nausea, emesis, diarrhea, abdominal pain), the renal system, neuromuscular (myalgia) and skin adnexa [26].

Clinical manifestation of infection is therefore extremely heterogeneous, ranging from completely asymptomatic or paucisymptomatic subjects to critically ill patients who require hospitalization and ventilatory support in intensive care unit [27, 28]. Since the beginning of the pandemic, the medical community has been aware of the greater susceptibility of patients with advanced age and comorbidities to the most serious forms of the disease, but we now know that patients with a younger age can also be critically affected [29].

Although virus-host interactions have been deeply investigated [30–32], the mechanisms underpinning a longer persistence of the symptoms in some patients or their recurrence (4 to 5 weeks, or even 1 year) after the resolution of the disease remain to be understood [33].

The persistence of fatigue, headache, and anosmia, the onset of anxiety and a depressive state are symptoms that have recently been included in the so-called post-acute (long) COVID-19 [26, 33, 34].

Like COVID-19, post-acute (long) COVID-19 is configured as a systemic disease and therefore symptoms are extremely varied and of difficult clinical interpretation. They can occur singly or in combination, they can be transient, intermittent or constant, and they can even change over the course of the condition.

The systems involved in post-acute (long) COVID-19 are mainly respiratory, musculoskeletal, cardiovascular and neurological [35].

Given the predominantly respiratory nature of the condition, lungs are the organs susceptible to the most severe outcomes, not only on a structural level (*e.g.*, secondary interstitial fibrosis, pulmonary hypertension) [36, 37], but also on a functional level (*e.g.,* reduced ventilatory capacity, dyspnea, fatigue) [37–39].

Respiratory sequelae have inevitably been shown to have repercussions at neuromuscular levels. In fact, dysfunctions of both respiratory and skeletal muscles have been described in about 40% of patients admitted to intensive care units, resulting in persistent symptoms of fatigue, weakness and shortness of breath [40–42]. Furthermore, it has been hypothesized that a direct muscle affection of SARS-CoV-2 may be responsible for structural alterations, even in patients who have had an apparently mild disease outcome [43].

The heart has been shown to be a target organ of the systemic inflammatory response and subject to direct damage from SARS-CoV-2 [35]. Specifically, the most described cardiovascular complications refer to heart failure, arrhythmias, peri-myocarditis, venous and arterial thromboembolism and “reverse Tako-Tsubo” cardiomyopathy [35, 44, 45].

Approximately 25% of patients who developed COVID-19 experienced neurological disorders of various degrees in the months following diagnosis [46]. The most common and mild symptoms include headache [47], disturbances in perception of taste and smell [48–50], “brain fog” and memory disorders [51]. Among the major complications, however, those mostly described were the presence of diffuse brain damage of inflammatory [52] or acute metabolic origins (toxic-metabolic encephalopathies) [53], Guillain-Barré syndrome [54, 55], Miller-Fisher syndrome [56], ischemic vasculitis [57], dysautonomic dysfunctions [55, 58, 59] and seizures. In addition to these complications. we urge the scientific community to deeply investigate mood disorders that might develop on a psychological substrate in response to stressors established during the pandemic period [46, 52, 60, 61]. Various mechanisms may underlie the neurological implications of SARS-CoV-2 [35, 62]. The systemic inflammatory response triggered in COVID-19 patients could potentially accelerate the evolution of neurodegenerative processes by exacerbating chronic conditions present at the time of infection but not yet manifest [35, 63, 64]. For example, the inflammatory response could exacerbate a refractory epileptic condition [65]. Several studies also reported direct damage of the brain tissue caused by SARS-CoV-2 infection [35, 66, 67].

In a novel, quantitative, longitudinal imaging study from Douaud et al. [68], authors analyzed brain scans of 401 SARS-CoV-2 positive cases, acquired at two time points (before and after testing positive for infection), and 384 matching controls. Following the description of hundreds of derived phenotypes, the comparison provided suggestions as to what could be further investigated as effect caused by, or attributable to, the SARS-CoV-2 infection. A clear involvement of the olfactory cortex has been detected through variations in tissue damage markers. The evaluation of gray matter decrease showed FDG (fluorodeoxyglucose) hypometabolism in the orbitofrontal cortex, insula, parahippocampal gyrus, and anterior cingulate cortex, suggesting a significant implication of the regions connected to the piriform cortex. In conclusion, the effect of reduction in the gray matter appears to be generalized, with a greater relevance at the level of the olfactory system.

However, to date, knowledge of the molecular mechanisms and neurological consequences in the post-acute (long) COVID-19 is still limited [69, 70].

In a broad sense, it has been hypothesized that post-acute (long) COVID-19 can be considered a condition characterized by a chronic persistence of low levels of inflammatory cytokines [71]. According to this assertion, it is likely that the activation of some cellular transcription factors, including the nuclear factor erythroid 2 (NFE2)-related factor 2 (Nrf2), a possible therapeutic target in several chronic neurodegenerative conditions [72–74], may have a role in increasing the expression of enzymes capable of synthesizing glutathione, therefore reducing the state of oxidative stress [71, 74, 75]. However, further data and trials are needed [76].

A recent study highlighted a significant formation of blood micro-clots, both in the acute phase and in the post-illness phase. These micro-clots seem to show resistance to the body’s fibrinolytic processes in patients suffering from post-acute (long) COVID-19. Preliminary results demonstrated the efficacy in reducing the symptoms of long COVID-19 patients through the administration of antiplatelet or anticoagulant therapy [77].

Understanding the signs and symptoms of the disease and of post-acute (long) COVID-19 represents a major current therapeutic challenge. This will allow us, in the near future, not only to better elucidate the molecular mechanisms of our body’s response to SARS-CoV-2 infection, but also to identify therapeutic targets for an increasingly personalized medicine.

## Genetic susceptibility in the host

Viruses, like other pathogens, are necessary, but not sufficient, to trigger disease [78]. It therefore appears evident that the individual host genome plays a fundamental role, not only in the susceptibility to disease induced by the infectious agent, but also in the individual response in terms of severity of the phenotype or resistance to infection [78]. Numerous host genes have been identified in the last two years that are active in susceptibility/resistance to infectious diseases [5, 13, 79–81]. Identifying and qualifying these genes as prognostic and predictive biomarkers is crucial to optimize patient management and promote sustainable and rational public health (PH) interventions. In addition, they contribute to clarify the mechanisms and variability of the SARS-CoV-2 host–pathogen interactions [81].

Common and rare variants have been identified in different studies using a) classical Genome-Wide Association Studies (GWAS) and b) deep sequencing of genes coding for protein referable to precise biochemical pathways involved in the pathogenesis of the infection. These studies have made it possible to identify alleles of increased susceptibility and/or partial resistance to the COVID-19, in coding and non-coding regions of genes. For example, a functional analysis of a SNP (rs11385942), identified by GWAS on chromosome 3p21.31, demonstrated the involvement of the LZTFL1 protein (leucine zipper transcription factor like 1) [82], which regulates ciliary localization in the BBSome complex. This gene is mutated in Bardet-Biedl Syndrome (BBS) (MIM#209,900), a ciliopathy characterized in part by polydactyly, obesity, cognitive impairment, hypogonadism, and kidney failure. *LZTFL1* is highly expressed in ciliated cells, including airway ciliated cells. Its reduced expression leads to fewer airway ciliated cells with shorter cilia, which could result in inefficient viral airway clearance in COVID-19 patients. Similarly, the SNP rs74956615, which maps on the chromosome 19p13.2 in the untranslated 3' of the *RAVER1* gene [82], has been found to modulate the expression of *RAVER1* itself. This gene encodes for a ribonucleoprotein which cooperates with cytoskeletal proteins vinculin/metavinculin and alpha-actinin to modulate alternative splicing events. However, *RAVER1* is a co-activator of MDA5 (IFIH1), which recognizes nucleic acids associated with viral infections such as dsRNAs, including SARS-CoV-2, and activates antiviral response genes, including *IFNB1*, *ICAM1*, *TNF* and *CCL5*. A large human genetic study, involving more than 49,000 COVID-19-affected individuals and 2 million control subjects, identified 13 loci in the human genome that affect COVID-19 susceptibility and severity including 6 loci previously not reported [83]. In the regions mapped by this extensive GWAS, authors identified more than 40 candidate genes, several of which are involved in immune function or have known functions in the lungs, suggesting that these may have important effects on COVID-19. A suggestive susceptibility locus on chromosome 12q22, has been recently detected in Thai population [84]. Genes mapped in this area include *EEA1* and *LOC643339*. *EEA1* is involved in viral entry into cells, while *LOC643339* is a long non-coding RNA. Intriguingly, *EEA1* is involved in the entry of African Swine Fever Virus via endosomal pathway [85]. Utilizing a Phenome-Wide Association Study (PheWAS) approach, Regan and Colleagues [86] identified novel phenotypic associations with genes encoding proteins active in the antiviral response and inflammatory processes. These genomic biomarkers can have pleiotropic effects in COVID-19-related comorbidities (cardiovascular disease, autoimmune disease, arthropathy and endocrinopathy), which in turn increase the risk of severe COVID-19.

Finally, a recent GWAS meta-analysis that considered 125,584 cases and over 2.5 million controls evaluating 60 studies from 25 countries included 11 new significant loci at the genome level, in addition to those previously identified. Genes in the new loci include *SFTPD*, *MUC5B* and *ACE2*, revealing convincing information on the susceptibility and severity of the disease [83].

Candidate-gene approach, on the other hand, made it possible to confirm and integrate the role of some specific pathways and proteins in the pathogenesis of the disease. Among the first candidate genes studied in SARS-CoV-2 infection were those coding for the HLA system, which plays a crucial role in the immune response [87, 88]. Several studies have highlighted risk alleles capable of influencing the clinical course of patients infected with various RNA viruses (e.g., H1N1 influenza virus [89], Hantaan virus [90] and SARS-CoV-1 [87]). Several studies have highlighted HLA alleles of susceptibility to SARS-CoV-2 [28, 91]. However, these studies revealed discrepancies due to different stratifications of patients and controls and to the different frequency distribution of the HLA alleles in the populations analyzed. Recently, a large and accurate study described a potential association of HLA-C*04:01 with severe clinical course of COVID-19. Carriers of HLA-C*04:01 had twice the risk of needing intubation when infected with SARS-CoV-2 [92]. Numerous other candidate genes have been analyzed on the basis of their biological function during infection, such as *ACE2*, *TMPRSS2*, *DPP4*, and Furin, involved in the entry of the virus into cells, or genes active in the viral egress such as *WWP1* and *NEDD4* [93–97]. Curiously, an association of *VDR* gene polymorphisms with COVID-19 outcomes has been also detected [98]. The possible involvement of this receptor is supported by recent studies that provided evidence for an altered vitamin D gene signature in CD4 + T lymphocytes in patients with severe COVID-19. Chauss and colleagues [99] demonstrated that severe COVID-19 may result from a dysfunction of type I immune response, that involves the vitamin D receptor (VDR) signaling. Similarly, it is interesting to observe how individuals with African descent, homozygous for the G1 or G2 variant of apolipoprotein L1 (*APOL1*), have an increased risk of acute kidney disease compared to subjects with low-risk variants [100]. A recent study revealed an association of phenotype severity and polymorphisms of the *MBL2* gene, which encodes a mannose-binding lectin (MBL) secreted by the liver and involved in innate immune defense [101]. Innate immunity is our immune system’s first line of defense and plays a central role in SARS-CoV-2 infection [102]. Although studied for over 100 years, only in recent years has significant progress has been achieved, largely due to the genetic dissection of innate immune pathways [103].

Several clinical and immunological studies have shown that type I interferons (IFN-I) play critical roles in the control and pathogenesis of COVID-19 [81, 104–107]. This notion is supported by extensive sequencing of numerous patients with severe forms of COVID-19 that identified pathogenic mutations in genes encoding active proteins in the interferon circuit [81]. The characterization of autoantibodies capable of neutralizing IFN-I in 10–15% of severe patients allows us to state that COVID-19 can be defined as an interferonopathy [108].

Identifying susceptibility alleles in COVID-19 is important in order to improve predictive testing and stratify different subgroups of SARS-CoV-2 positive subjects, which can be treated in a personalized way. However, it is possible that in a complex multifactorial and multigenic disease, such as COVID-19, several genetic, epigenetic and socio-demographic factors are modulating the phenotypic manifestation, thus complicating the analysis of genotype–phenotype correlations [32].

Interestingly, the CHGE Consortium (Covid Human Genetic Effort, https://www.covidhge.com/about) initiated a study to enroll individuals (referred to as “resistant”) who were not infected with SARS-CoV-2 despite repeated exposure (*e.g.*, care-givers or familiars of a patient with severe pneumonia), as evidenced by the absence of the disease and virus specific antibody titers in several tests [81, 106, 108–111]. It is conceivable that these subjects carry monogenic variations that make them naturally resistant to virus entry, or much more active in eliminating the virus by activating appropriate defense mechanisms such as the genes of the interferon circuit. Interestingly, a splice variant of *OAS1* gene, which appears to have a protective effect, has been identified frequently in people of African ancestry [112]. *OAS1* encodes for an enzyme catalyzing the synthesis of short polyadenylates, which activate ribonuclease L that in turn degrades intracellular double-stranded RNA and triggers several other antiviral mechanisms [113].

Using trans-ancestry fine-mapping approaches, Huffman et al. [114] recently demonstrated that the rs10774671-G splice variant determines the length of the protein encoded by the gene *OAS1,* which results in an enzyme more effective at breaking down SARS-CoV-2. It is important to clarify that the genes of susceptibility to pathogens, although of biological and genetic interest, cannot in any way confer a sort of “natural immunity” to infection at an individual level and cannot replace the important protective role offered by vaccines.

Certainly several “resistance” alleles of genes involved in the different pathways activated by the infection of SARS-CoV-2 will be identified in the next months [80, 95, 112]. However, until it is possible to develop polygenic scores programs which must then be validated on large numbers, it is unlikely that they can be used to identify resistant subjects and direct them to selective and specific therapeutic treatments. These studies have made it possible to elucidate many aspects of the pathogenesis of COVID-19 and have provided many biological responses to the pathogen-host relationship that could prove important in other viral infections [81]. In this regard, it seems interesting to report a recent study that correlates the loss of smell or taste, very frequent in COVID-19, to variants of the *UGT2A1* and *UGT2A2* genes expressed in the olfactory neuroepithelium, which lines the posterior nasal cavity, and is exposed to a wide range of odorants and compounds present in the air [115].

**Fig. 2:**
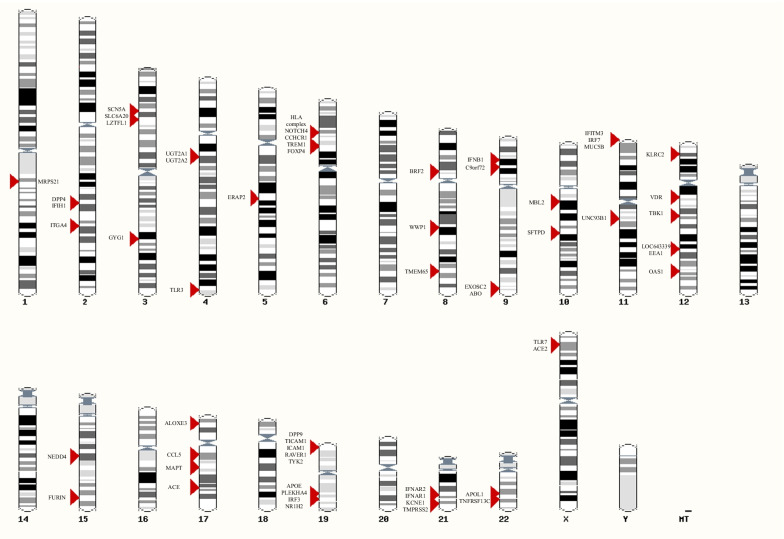
Chromosome ideogram representing the location of genes of interest investigated for a role in defining susceptibility to SARS-CoV-2 infection (generated by ensembl.org [116])

The locations of investigated genes of interest are resumed in Fig. 2 [5, 116].

## Characteristics of available vaccines

While witnessing an exponential progress in studies aimed at understanding genetic and molecular mechanisms, we see their direct application in the tools which are currently the best candidates to lead us out of this pandemic: vaccines.

Since the beginning, several critical issues emerged which led to base the development of vaccines on safety, immunogenicity, durability of the immunity, dosing schedule, technological platform and ease of manufacture and transport.

Despite a widespread mistrust about safety and speed of production, nowadays, we can benefit of two types of vaccines against SARS-CoV-2 and of a growing number of data that support their efficacy and safety. Two messenger RNA (mRNA) (BNT162b2 and mRNA-1273) and two viral vector (ChAdOx1 nCoV-19 AZD1222 and Ad26.COV2.S) COVID-19 vaccines were developed [117]. A third type, protein subunit vaccines (NVX-CoV2373), has been approved by EMA, Indonesia, Australia and South Korea, while is still lacking a full approval by the FDA.

Findings show that the Pfizer/BioNTech BNT162B2 vaccine is safe, with very rare incidence of myocarditis and swelling of the lymph nodes while coronavirus infection is associated with numerous serious adverse events such as increased risk of pericarditis, arrhythmias, heart attacks, strokes, pulmonary embolism, deep-vein thrombosis, acute kidney damage, and others [118]. The BNT162b2 COVID-19 vaccine has been shown to reduce viral load of breakthrough infections (BTIs), but its effectiveness declines after the third to fourth month [119].

The appearance of new SARS-CoV-2 variants poses new challenges for the development of vaccination platforms [120]. As a consequence, mRNA booster vaccines were developed to restore the viral neutralization activity that wanes after the initial two-dose vaccination, to maintain protection against emerging variants and to increase vaccine effectiveness in low immune response individuals such as elderly or immune suppressed. The need for multiple doses of the vaccine has sparked new debates, but evidence shows that vaccination with two doses of mRNA-1273 (Moderna) and a booster are safe and effective [121]. Moreover, the effectiveness of a third BNT162b2 vaccine booster was demonstrated in both reducing transmission and severe disease [122].

As previously stated, we are now aware that higher age and comorbidities are risk factors for poor outcomes, regardless of vaccination status [123, 124].

Among adolescents aged 16–17 years, 2-dose mRNA vaccine effectiveness increased to 86% a week days after booster dose and urgent care hospitalizations were substantially lower during the Omicron period than during the B.1.617.2 (Delta) predominant period among adolescents aged 12–17 years, with no significant protection ≥ 150 days after dose 2 during Omicron predominance [125]. An increasing number of studies are focusing on the efficacy of multiple doses in fragile categories: cancer patients receiving at least two doses of COVID-19 vaccine show reduced risk of COVID-19 [126]. Despite diffused concerns, it has now been established that the BNT162b2 and Ad26.COV2.S vaccines can be safely administered during the third trimester of pregnancy, reporting excellent results in terms of immunogenicity [127].

The majority of vaccinated patients who required hospitalization due to COVID-19 were elderly with a high comorbidity burden thus being unable to develop a proper immune response following vaccination [128].

CDC recommends that all persons aged 12 years and older receive a booster dose of COVID-19 mRNA vaccine at least 5 months after completing the primary vaccination series (at least 2 months after receiving J&J/Janssen COVID-19 vaccination) and that adults 50 years and older, and moderately or severely immunocompromised people, if eligible, should receive a second booster dose at least 4 months after the previous one.

Vaccines impact on containing the pandemic escalation is evident [129]. Their effectiveness is clearly dose-dependent as it is higher after administration of a third dose compared to a second dose administration; however, vaccine effectiveness wanes with time [129]. For this reason, and because of the emerging variants that might overtake the currently available vaccines, the development of new vaccines, including variant-specific ones, should be encouraged and strongly supported. As of April 15, 2022, 153 vaccines are listed in clinical development and 195 in pre-clinical development (WHO, https://www.who.int/publications/m/item/draft-landscape-of-covid-19-candidate-vaccines, accessed on April 15, 2022) [130].

## Outlook

At the official age of about 3 years, SARS-CoV-2 is no longer a baby, but it has proven a rather vicious toddler. Although ascribing intentions to a virus is naively anthropomorphic, we have to admit that it has managed to cut short the lives of many fellow-human beings, to change our ways of relating to each other, to subvert economies, to shift the priorities of pharmaceutical industry and of regulatory bodies. With respect to biomedical and public health research, as of May 29, there are 262,077,248 papers on COVID-19 listed in *PubMed*, but later tonight there will be more; and when we wish to discuss science, we pretend that meeting on line is just as good as being together in a seminar room – but it is not true.

From the evolutionary point of view, it has been a long-held tenet of parasitology that for a parasite what is at a premium is not to kill the host; rather, to have the host producing the maximum amount of parasite progeny. SARS-CoV-2 is a perfect illustration. The people infected have been at least half a billion: Mortality has been therefore high in absolute numbers, but at least 98% infected people have survived and have helped to spread the virus. Since the beginning of the pandemic, there have been thousands of mutations in the virus, most of them biologically neutral; at the moment, the predominant Omicron variant seems to be a compromise between high infectivity and relatively low mortality: Seen from the vantage point of the virus, the compromise is good, but not necessarily optimal as yet.

The wealth of studies that have explored the genetic variation in host susceptibility to SARS-CoV-2 is impressive. There seem to be genetic factors that affect the probability of contracting the infection; and many studies have naturally preferred to focus on genetic factors that may allow the disease to become severe and life-threatening. We must admit that we have not yet hit the jackpot: Nobody has found a human genotype that bars infection, as homozygosity for the delta 32 allele of the *CCR5* gene does for HIV [131]. Perhaps this is not altogether surprising: For an infectious agent to select a protective gene, it requires exposure to be continuous over the host’s many generations [132], as has been the case for *P falciparum* and the hemoglobin S gene [133]. If the postulated jump of SARS-CoV-2 into the human species has been very recent, there has been no time for selection of people having an originally rare mutant gene.

In last year’s update [13], we did briefly speculate about the apparently low numbers of COVID-19 cases in tropical Africa, and according to the WHO dashboard, this is still the case. In the two countries, we know best, there have been, officially, in Nigeria (population over 200 million) 119,322 cases and 1926 deaths to date; in Tanzania (population around 60 million), which for over a year has not reported cases, there are now on record 33,726 cases and 800 deaths. We do not yet know to what extent these low figures are due to underestimation; but from recent visits, we know that health workers are vaccinated. As for people in general, what we have learned to my surprise is that vaccine availability is not currently a limiting factor: Even when vaccination is free, uptake is quite low.

Anti-COVID-19 vaccination has been a success of technology and, in many countries, of public health campaigns. Some of us never thought that an effective vaccine could be designed, produced and field-tested within one year: but we have to stand corrected. In retrospect, the notion of using RNA to immunize against an RNA virus seems straightforward: Ugur Sahin and Ozlem Tureci deserve full scientific credit (in addition to many € millions in revenue) for what they have achieved. With most previous vaccines, peptides derived from the organism or from the purified protein injected are presented to T cells by Antigen Presenting Cells (APC). In this case, instead, nanoparticles that encapsulate the portion of the viral RNA that encodes the spike protein are endocytosed by the APCs, whose protein synthetic machinery is taken over to translate the RNA into the spike protein, peptides from which are then presented to T cells. A key to the ultra-fast development of the BioNTech vaccine has been this clever approach: Since it was unprecedented, nobody was entitled to predict (i) How it would work in practice, (ii) What would be the duration of immunity. With respect to (i) The results have been spectacular; with respect to (ii) One had to find out the answer empirically, and it turned out that, even after two doses, the duration is of the order of months, not years. Only the oldest among us can remember that when mRNA was discovered, one of its defining properties was a short life span [134]: It seems not far-fetched to hypothesize that the takeover of the APC’s ribosomes by SARS-CoV-2 mRNA is short-lived, and this may be at least one reason why immunity does not last very long.

Unlike the COVID-19 epidemic, the epidemic of no-Vax does not lend itself to mathematical analysis: It is not in the realm of biological science, but rather in the realm of psycho-patho-sociology [135]. We have all learnt that evidence-based reasoning does not make a dent in the hard-core no-Vax. Confrontation fails, and an approach based on lateral thinking may be better. Perhaps we should take this on as an intellectual challenge: If we can largely prevent severe COVID-19 by immunization, if we can find the molecular basis of many diseases, if in many cases we can cure leukemia, we should be able to also address the no-Vax problem.

## Data Availability

Data sharing is not applicable to this article as no datasets were generated or analyzed during the current study.

## Abbreviations

ACE2: Angiotensin-converting enzyme 2
APC: Antigen presenting cells
APOL1: Apolipoprotein L1
BBS: Bardet-Biedl syndrome
CCL5: CC-chemokine ligand 5
CDC: Centers for disease control and prevention
CHGE: Covid human genetic effort
COVID-19: Coronavirus disease 2019
CSSE: Center for systems and software engineering
CT: Computed tomography
DPP4: Dipeptidyl peptidase 4
EEA1: Early endosomal antigen 1
EMA: European medicines agency
FDA: Food and drug administration
FDG: Fluorodeoxyglucose
GWAS: Genome-wide association study
H1N1: Hemagglutinin type 1 and neuraminidase type 1
HLA: Human leukocyte antigens
ICAM1: Inter-cellular adhesion molecule 1
IFN: Interferon
IFNB1: Interferon beta 1
LZTFL1: Leucine zipper transcription factor like 1
MBL: Mannose-binding lectin
MDA5: Melanoma differentiation-associated rotein 5
MUC5B: Mucin protein 5B
NEDD4: Neural precursor cell expressed developmentally down-regulated protein 4
NFE2: Nuclear factor erythroid 2
NRF2: Nuclear factor erythroid 2 (NFE2)-related factor 2
OAS1: 2'-5'-Oligoadenylate synthetase 1
PH: Public Health
PheWAS: Phenome-wide association study
RAVER1: Ribonucleoprotein PTB-binding 1
SARS-CoV-2: Severe acute respiratory syndrome coronavirus-2
SFTPD: Surfactant protein D
SNP: Single nucleotide polymorphism
TMPRSS2: Transmembrane serine protease 2
TNF: Tumor necrosis factor
UGT2A: UDP-glucuronosyltransferase 2A
VDR: Vitamin D receptor
VOC: Variant of concern
WHO: World health organization
WWP1: WW domain containing E3 ubiquitin protein ligase 1
